# Single Cell Confocal Raman Spectroscopy of Human Osteoarthritic Chondrocytes: A Preliminary Study

**DOI:** 10.3390/ijms16059341

**Published:** 2015-04-24

**Authors:** Rajesh Kumar, Gajendra P. Singh, Kirsten M. Grønhaug, Nils K. Afseth, Catharina de Lange Davies, Jon O. Drogset, Magnus B. Lilledahl

**Affiliations:** 1 Department of Physics, Norwegian University of Science and Technology (NTNU), N-7491 Trondheim, Norway; E-Mails: catharina.davies@ntnu.no (C.L.D.); magnus.lilledahl@ntnu.no (M.B.L.); 2 Physical Optics and Electronics Group, Massachusetts Institute of Technology (MIT), Cambridge, MA 02139, USA; E-Mail: gpsingh@mit.edu; 3 Orthopaedic Department, Levanger Hospital, Kirkegata 2, N-7600 Levanger, Norway; E-Mail: kirstengroenhaug@gmail.com; 4 Nofima, Postbox 210, N-1431 Ås, Norway; E-Mail: nils.kristian.afseth@nofima.no; 5 Department of Orthopaedic Surgery, Trondheim University Hospital, N-7491 Trondheim, Norway; E-Mail: jon.o.drogset@ntnu.no

**Keywords:** Raman spectroscopy, osteoarthritis, chondrocytes, biomedical optical diagnosis

## Abstract

A great deal of effort has been focused on exploring the underlying molecular mechanism of osteoarthritis (OA) especially at the cellular level. We report a confocal Raman spectroscopic investigation on human osteoarthritic chondrocytes. The objective of this investigation is to identify molecular features and the stage of OA based on the spectral signatures corresponding to bio-molecular changes at the cellular level in chondrocytes. In this study, we isolated chondrocytes from human osteoarthritic cartilage and acquired Raman spectra from single cells. Major spectral differences between the cells obtained from different International Cartilage Repair Society (ICRS) grades of osteoarthritic cartilage were identified. During progression of OA, a decrease in protein content and an increase in cell death were observed from the vibrational spectra. Principal component analysis and subsequent cross-validation was able to associate osteoarthritic chondrocytes to ICRS Grade I, II and III with specificity 100.0%, 98.1%, and 90.7% respectively, while, sensitivity was 98.6%, 82.8%, and 97.5% respectively. The overall predictive efficiency was 92.2%. Our pilot study encourages further use of Raman spectroscopy as a noninvasive and label free technique for revealing molecular features associated with osteoarthritic chondrocytes.

## 1. Introduction

Osteoarthritis (OA) is a complex musculoskeletal disorder. It is a leading cause of disability among elderly people and is a major public health issue with increasing individual and socioeconomic burden. It was recently found that it is the fastest increasing major health condition in terms of the global rank: “Years lived with disability (YLD)” [1]. Although the use of the Kellgren-Lawrence (K/L) score obtained by radiography is a widely accepted method for the diagnosis of OA, several studies have demonstrated the complexity involved in early-stage diagnosis [2,3,4].

The origin of OA is not exactly clear and the understanding about the etiology of OA is continuously evolving. There are several reports which review the changes observed during progression of OA [5,6]. It is believed that the disease primarily affects the articular cartilage and the associated underlying bone. The prominent histological changes in cartilage are erosion, fibrillation, clefts, chondromalacia, loss of metachromasia, duplication of tidemark and cloning of chondrocytes. The underlying cause of these macroscopic and microscopic structural features is the change in biochemical compositions such as alteration and decrement in content of proteoglycan [7]. Biochemical changes are triggered by expression of enzymes, which are responsible for the breakdown of matrix components. It is believed that expression of such enzymes occurs due to the imbalance in cell homeostasis, *i.e.*, increased catabolic activity by chondrocytes [8]. Several studies also show that progression of OA is associated with increased chondrocyte cell death [6,9,10]. Investigations at the cellular level are, therefore, important in understanding the progression of OA as well as other cartilage disorders.

Raman spectroscopy has recently attracted attention because it shows the potential for minimally invasive, label free, and objective diagnostics at molecular level. The advantage of a label free technique is that it provides the information from the target molecules that are not modified. Therefore, all processes in the target molecules are undisturbed and the cells do not show a stress reaction attributable to an external label [11]. Raman spectroscopy is a laser based vibrational spectroscopic technique and has numerous times successfully demonstrated early detection of neoplasia, differentiation of tumors grades, and been useful in providing sub-cellular information from the living cells [12,13]. However, to the knowledge of author, this is the first report on Raman spectroscopy of osteoarthritic human chondrocytes. The aim of this pilot study is to demonstrate the capabilities of Raman spectroscopy for single cell analysis in order to identify major changes in the spectroscopic signatures of the cells during the progression of OA. In addition, we have demonstrated that based on biochemical compositions at cellular level, Raman spectroscopy is capable to distinguish the chondrocytes isolated from different stages (ICRS Grade-I, II and III) of osteoarthritic cartilage.

## 2. Results and Discussion

**Figure 1:**
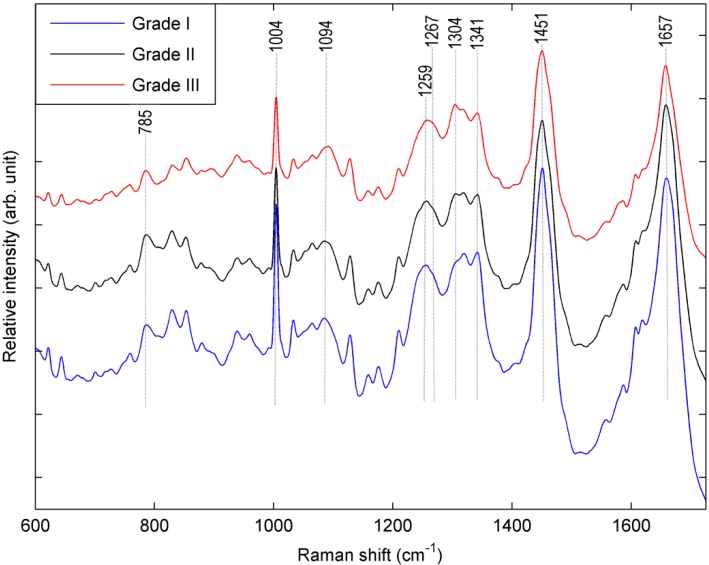
Normalized mean (*n =* 150) Raman spectra of chondrocytes which were isolated from osteoarthritic cartilage of ICRS Grade-I, II and III. Spectra are vertically offset for clarity.

A comparison between the mean (of *n =* 150) spectra of ICRS Grade-I, II and III is shown in Figure 1. Mean spectra represent the average characteristics of each grade. Typically, Raman spectra obtained from biological samples are complex in nature, consisting of superimposed Raman peaks associated with the various bio-molecular constituents of the cell. Hence, visual interpretation of the mean spectra obtained from a large variety of cells is not straightforward. However a careful qualitative evaluation of the mean spectra can provide an insight into the bio-compositional changes at the cellular level during the progression of OA. In order to confirm that the subtle differences involved in the spectra are from variation of the Raman signals and not from the fluorescence background, the variance between the mean spectra of the three different cell types (Grade I, II and III) at each wavelength positions was plotted (Supplementary Figure S1). The plot of variance *vs.* wavelength expressed major Raman peaks (and not fluorescence background) associated with cellular biochemical components which are described in next section.

**Figure 2:**
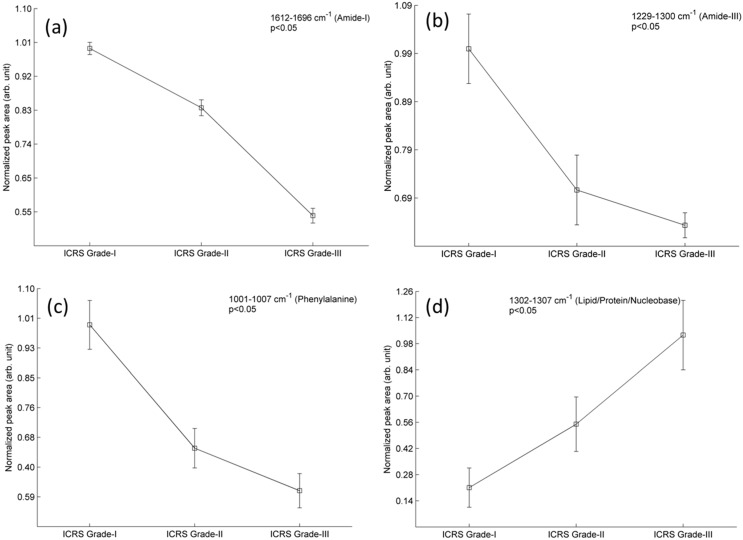

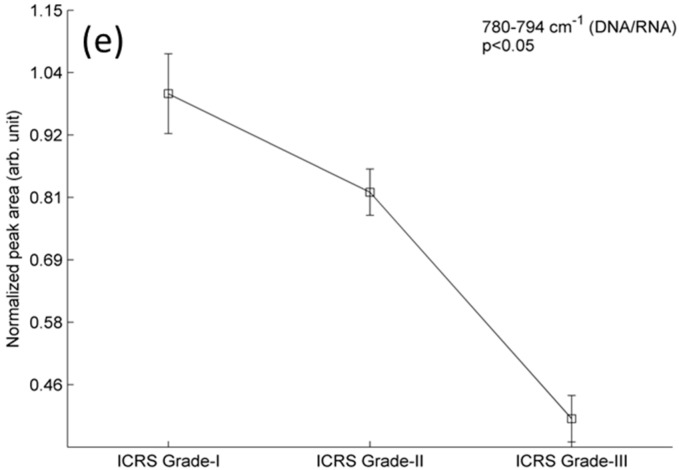
A relative comparison for the content of amide-I (**a**); amide-III (**b**); phenylalanine (**c**); lipid (**d**); and DNA/RNA (**e**) in osteoarthritic chondrocytes (*n =* 150 cells). These chondrocytes were isolated from osteoarthritic cartilage section of ICRS Grade-I, II and III. Data represents the normalized mean area of Raman band and bar represents the standard error.

The main aim of this analysis was to investigate if Raman spectroscopy could be used to identify biomolecular changes at the cellular level and potentially discriminate chondrocytes which were isolated from different stages (ICRS Grade-I, II and III) of osteoarthritic cartilage. The major spectral changes in chondrocytes obtained from different stages of osteoarthritic cartilage can be found by a qualitative analysis of the mean spectra (Figure 1). The Raman spectra clearly indicate changes in protein vibrations. The magnitude of the area (Figure 2a) represented by amide I (1612–1696 cm^−1^) decreases consistently with an increase in the grade of OA. This observation was supported by similar trend (Figure 2b,c) shown by the content of amide III (1229–1300 cm^−1^) and phenylalanine (1001–1007 cm^−1^/peak at 1004 cm^−1^). These observations indicate a general decrease in protein content inside chondrocytes in higher grades of OA. Moreover, decrease in protein content was observed with decrease in the content of the nucleic acid (780–794 cm^−1^/peak at 785 cm^−1^) in cells which may correlate with the enhancement of internucleosomal DNA cleavage [14] during progression of disease (Figure 2e).

As another observation, intensity of the spectral band 1302–1307 cm^−1^/peak at 1304 cm^−1^ is increasing with progression of OA (Figure 1 and Figure 2d). This peak may be attributed to the lipids [15,16,17,18,19]. In the earlier reports, it was hypothesized that increased lipid deposition may be associated with the advancement of OA [20,21,22,23]. Our observation indicates that this peak could be an indicator of lipid deposition and hence, supports the earlier hypothesis that lipid deposition may play a role in progression of OA. However, other alternative interpretation (e.g., amide III and/or nucleobase) besides lipids might also be possible relevant to the peak of 1304 cm^−1^ [15]. Therefore, more experimental studies are necessary to verify the contribution of lipid during progression of the disease.

**Figure 3:**
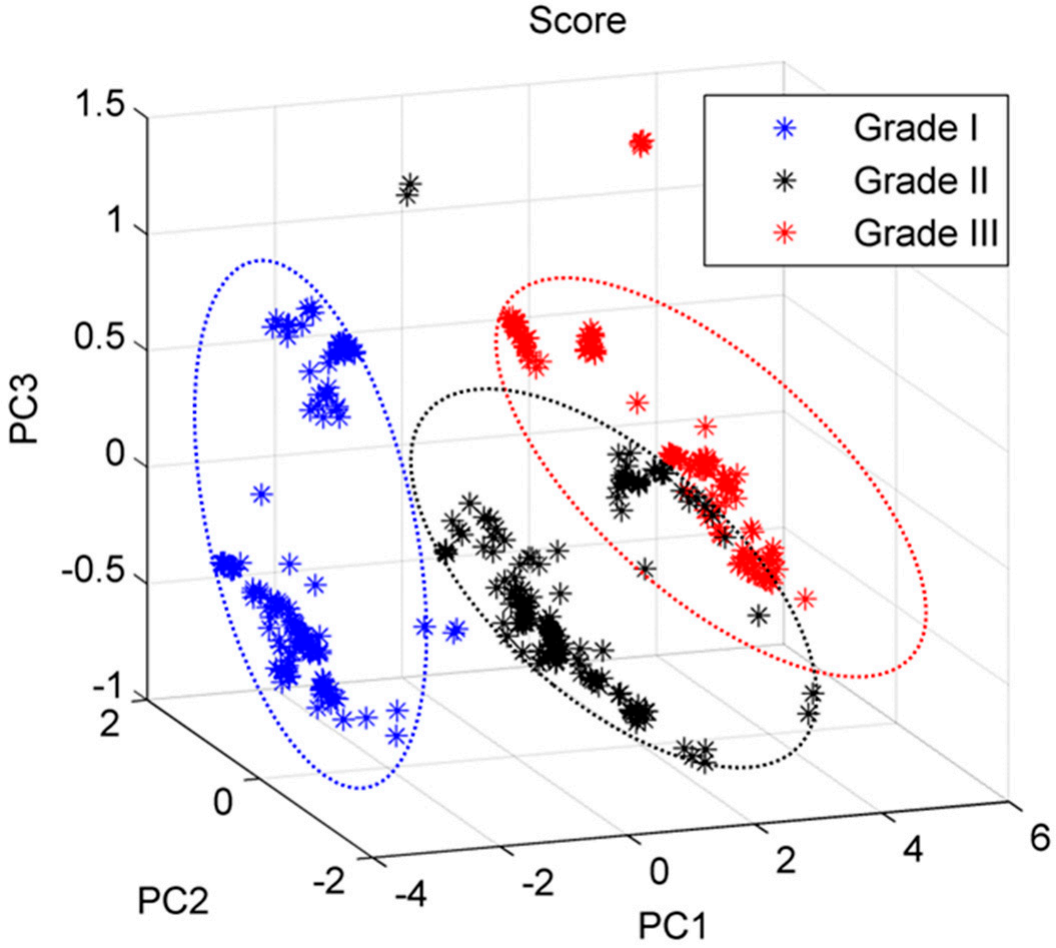
Multivariate-analysis based principal component analysis (PCA) algorithm classifies different osteoarthritic chondrocytes cells associated with ICRS Grade I, II and III into separate clusters.

In order to test the capability of Raman spectroscopy, spectra were compared objectively by applying multivariate analysis technique, using ICRS as a gold standard. To elucidate and visualize the hidden biochemical information, principal component analysis (PCA) was performed on the total data-set which were acquired from 450 samples (cells). PCA is a common multivariate statistical method that is often used in bioanalytical Raman spectroscopy to reduce dimensionality and to identify combinations of the most important spectral markers which maximizes the data variance and optimizes group separation [24,25]. It is performed over the data sets with the aim of comparing spectral data in terms of similarities and differences in an unsupervised manner [26]. The results of the PCA analysis are shown in Figure 3. The spectra of chondrocytes associated with the three different ICRS grades of OA appeared as distinct clusters when plotted against the three main PCs that explain maximum variance in the data set. The tightness and separation of the clusters illustrates how easily the analytes can be discriminated. In order to quantitatively assess discrimination capability, prediction accuracy was tested subsequently using leave one out full cross-validation technique using Mahalanobis distance as a discriminator. Several reports recommend full cross-validation as a general approach to prediction testing, claiming that this should provide most comprehensive testing of the model [27,28]. The classification matrix summarizes the correct and incorrect classification of the spectra (Table 1). The specificities for ICRS Grade I, II and III were 100.0%, 98.1%, and 90.7% respectively, while, the sensitivities were 98.6%, 82.8%, and 97.5% respectively. The overall predictive efficiency was 92.2%. Additionally, the segmented [27] cross-validation (a segment of 10 spectra at a time) showed similar result in terms of overall predictive efficiency (91.8%). Our analysis shows that the chondrocytes obtained from grade-I osteoarthritic cartilage, were classified with higher accuracy than other grades. Misclassifications mainly occurred between the chondrocytes of grade II and III (Figure 3), which indicates that chondrocytes associated with ICRS grade II and III are more heterogeneous in biochemical compositions than ICRS Grade-I. During progression of OA the quality of articular cartilage degrades and chondrocytes respond in order to cope with physiochemical changes occurred in extracellular matrix [29,30]. In this context, our result indicates that to a greater extent most chondrocytes available in ICRS Grade-I cartilage are in similar stage, but, the partial overlap between the cluster of ICRS Grade-II and Grade-III indicate that all chondrocytes associated with macroscopically higher grade (*i.e.*, Grade-II and III) of osteoarthritic cartilage are not in same stage of degradation.

**Table 1:** Classification matrix provides the information about classification ability and prediction efficiency of PCA algorithm.

Sample: Chondrocytes	Grade-I	Grade-II	Grade-III
Grade-I (150)	150	0	0
Grade-II (150)	2	145	3
Grade-III (150)	0	30	120

**Figure 4:**
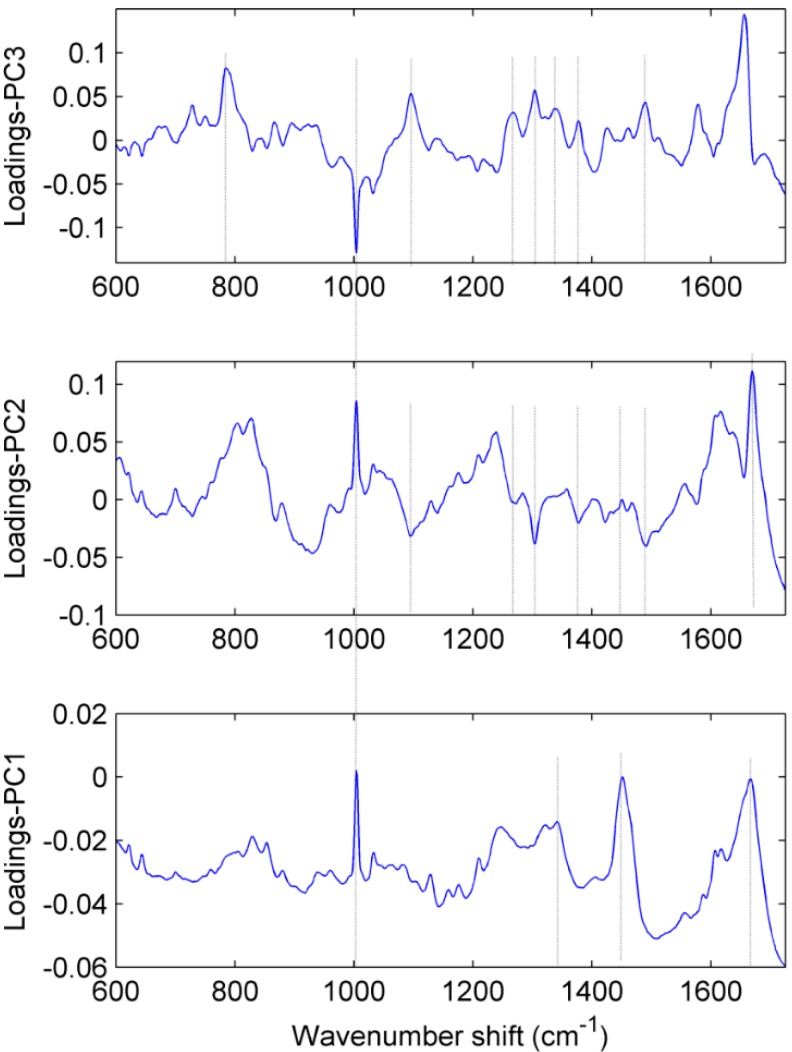
Loading plots relevant to PC1, PC2 and PC3, which are mainly responsible for the discrimination among different clusters of chondrocytes cells associated with different grades of osteoarthritic cartilage.

Loading vector is associated to the original Raman spectrum by a variable called PC score, which represents the weight of that particular component against the basis spectrum [31]. Loading vectors (Figure 4) associated with three main principle components (PC-1, PC-2 and PC-3) were plotted. These plots are mainly composed of Raman peaks, which indicate that the major variations in the data are due to Raman signals and not fluorescence background. Loadings associated with PC-1, PC-2 and PC-3 explains 82.30%, 7.06% and 3.75% of the total variance in the data set, respectively. Combined, these three PCs explain 93.11% of the total variation in the data set. The most important spectral features which appeared in the loading plots were tentatively assigned [15,18] to amide-I (1657 cm^−1^), protein and carbohydrate (1451 cm^−1^), DNA/RNA (1341 cm^−1^), lipid and protein (1304 cm^−1^), amide-III (1267 cm^−1^), nucleic acids (1094 cm^−1^), phenylalanine (1004 cm^−1^) and DNA/RNA (785 cm^−1^). However, a few peaks (e.g., 1379 and 1490 cm^−1^) are remained unassigned for human cells and require further investigation.

Fixation of cells may induce spectral changes. Acetone and alcohol (e.g., methanol and ethanol) dissolve lipids. Therefore, other fixatives like formalin and 4% paraformaldehyde was considered as a better option for the fixation. As shown in previous spectroscopic studies using cultured cells that formalin fixation was found to affect the cellular lipid and protein content [32,33]. Ten percent formalin with fixation times of several hours are considered much harsher conditions than 4% paraformaldehyde that could lead to dehydration due to fixation [34]. Although fixation with 4% paraformaldehyde also induces changes in the Raman spectrum of a cell, the induced changes are small compared to the alcohol [34]. Moreover, with longer duration of fixation time, larger spectral changes were observed [34]. Therefore, 4% paraformaldehyde with mild fixation time (~10 min) was chosen to be optimal for the Raman study. However, additional studies are needed to find the fixation induced Raman changes due to the 4% paraformaldehyde especially in the chondrocytes.

Due to erosion and thinning in high grade OA [35,36,37], it is difficult to isolate the chondrocytes from a given cartilage layer (for example, superficial layer). Therefore, spectra were acquired from a large number of randomly chosen cells that were isolated from the whole volume (bulk) of the osteoarthritic cartilage. Hence, the spectra represent the chondrocyte characteristics of the bulk cartilage rather than a specific layer of cartilage. Thus, the spectral differences are associated with biochemical difference in OA severities (and not only the chondrocyte characteristics associated with different cartilage layer).

Due to ethical considerations, biopsies of normal (healthy) cartilage from human knee were not obtained in this study. Although our study includes the detection of osteoarthritic grades at cellular level, it should be noted that clinical diagnosis of OA is not performed at cellular level. Biopsy of early stage cartilage and then, isolation of chondrocytes for diagnostic purposes is not intended to be performed in clinical practice. Nevertheless, detailed knowledge of OA at cellular level is highly desirable. Enhanced understanding will help in exploring underlying mechanism and thus, in early diagnosis that may lead towards correct treatment and prevention of OA.

## 3. Materials and Methods

### 3.1. Human Osteoarthritic Cartilage Sections

The use of human samples was approved by the Regional Committee for Medical Research Ethics (2013/265 REK, Norway). Human articular cartilage samples were obtained from the femoral condyle of osteoarthritic patients undergoing total replacement knee surgery. In order to consider the case of primary OA, only those patients that had not suffered previous knee injury and had not undergone other prior surgery were included in the study. To reduce age-associated variation in the articular cartilage, all patients selected in this study were older than 65 years. It should be pointed out that different grades of osteoarthritic cartilage may be present within one femoral head of the knee joint. The grading of cartilage sections was based on the standard (International Cartilage Repair Society) ICRS classification (Supplementary Table S1) [38,39]. The assignment of ICRS grade to the cartilage sections was performed by two experienced orthopedic surgeons, who were blinded to the classification of each other. Only sections assigned a similar ICRS grade by both orthopedic surgeons were included in this study. In this pilot study, 15 specimens in total, 5 specimens of each ICRS Grade (Grade I, II and III) were included (for details please refer to Supplementary Figure S2). All tissue sections included in this study were obtained from load bearing area (medial) of the femoral condyle of the knee. Only osteoarthritic cartilage specimens of ICRS Grade I, II and III, but not Grade-IV, were included in the study. As described in the ICRS classification scheme, at the location of ICRS Grade IV (*i.e.*, advanced stage of OA), underlying bone is exposed and almost no cartilage present. Therefore, ICRS Grade-IV specimens were not included in this study.

### 3.2. Isolation of Chondrocytes (Cells)

Isolation of chondrocytes from all osteoarthritic cartilage specimens was performed by enzymatic (collagenase) digestion process. A typical image of osteoarthritic cartilage and isolated single cells is shown in Figure S3. To isolate chondrocytes, firstly, cartilage sections were divided into several pieces (~1–2 mm) using a surgical scalpel. Incubation of trimmed cartilage was then performed for ~18 h in 2–5 mL of Dulbecco’s Modified Eagle’s Medium (DMEM) containing 0.8 mg/mL collagenase from clostridium histolyticum (C6885; Sigma-Aldrich, Saint Louis, MO, USA) and 50 μg/mL gentamicin (G1397; Sigma-Aldrich). Filtration of the digested cartilage was performed through a 50 μm filter. Centrifugation of the filtered cells was performed at 1500 rmp for 5 min. Pelleted cells were resuspended in phosphate buffer saline (PBS) solution. Centrifugation and resuspension in PBS was repeated second time. Finally, cells were fixed by 4% pararfomaldehyde for the time duration of 10 min. Cells in PBS suspension were stored at 4 °C.

### 3.3. Raman Spectroscopy

Raman spectra were acquired using a standard confocal Raman microscope (LabRam HR800, HORIBA Jobin Yvon, Villeneuve-d'Ascq, France). The 632.10 nm laser light was focused by an Olympus (Tokyo, Japan) 60×, 1.2 NA water-immersion microscope objective. Cells sedimented in a well on a CaF_2_ slide and were covered by a 100 µm quartz cover-slip. 30 Raman spectra were acquired from 30 randomly chosen cells associated with each specimen (Supplementary Figure S2). Therefore, 150 (30 × 5 specimens) spectra were collected from each ICRS grade (Grade I, II, and III). In total, 450 (150 × 3 Grades) spectra obtained from 450 samples (cells) were used in this investigation (Figure S2). Spectra were acquired over the Raman shift region 600–1725 cm^−1^ (fingerprint region). The acquisition time of each spectrum was 90 s. During acquisition of data, the laser power at the surface of the sample was 8 mW. In order to minimize biochemical heterogeneity of different cell compartments (e.g., nucleus, cytoplasm, membrane), during each data acquisition, the laser was focused in the nucleus of the cell. However, due to inherent features such as multiple scattering and the large size of the confocal volume [40,41] the system might also partially examine the perinuclear structures. As described by Pudlas *et al.* [42], the collected Raman signals predominantly represents molecular bonds of the cell nuclei. In order to reduce the fluorescence background and enhance the comparability of the spectra, the raw Raman spectra were pre-processed by removal of cosmic rays (narrow noise spikes), subtraction of background spectra (the corresponding spectra of PBS suspension which were acquired after the change of every focus) and smoothing (Savitzky-Golay filter: 3rd order, 9 point). Spectra were then subjected to extended multiplicative signal correction (EMSC) [43], followed by peak normalization (1004 cm^−1^). The plots for the normalized mean (*n =* 150) area of the corresponding Raman band of interest were plotted with standard error. Spectral band assignment of sharp peaks (*i.e.*, 785, 1004 and 1304 cm^−1^) for the calculation of peak area was performed according to full width at half maxima (FWHM). Statistical comparisons were performed using one-way analysis of variance (ANOVA) and a *p*-value of less than 0.05 was considered as statistically significant. The data analysis was performed in Matlab (The MathWorks, Natick, MA, USA).

## 4. Conclusions

In summary, as a proof-of-principle study, we demonstrated Raman spectroscopy as a noninvasive and label free technique that can detect subtle biomolecular changes in osteoarthritic chondrocytes. The relative changes in protein, lipid and RNA content in chondrocytes at different stages of OA may help in exploring the underlying mechanism of the disease at cellular level. Additionally, we have shown that with high specificity and sensitivity, it can discriminate the cells obtained from the different stages of osteoarthritic cartilage. Our analysis might be helpful in the development of Raman spectroscopy technique to monitor OA progression at the cellular level. Further correlation and validation of novel Raman spectral features of chondrocytes using supervised/unsupervised techniques in combination with clinical gold-standard may help Raman spectroscopy to achieve its full potential. Nevertheless, our study encourages further investigations; for example: characterization of proteins [44,45], mapping of single cells [19,46,47], quantitative comparison of biochemical compositions (e.g., DNA/RNA, lipid, collagen, total protein content) [48] in different stage osteoarthritic chondrocytes, which may further reveal hidden features associated with progression of OA at cellular and sub-cellular level.

## Supplementary Materials

Supplementary materials can be found at http://www.mdpi.com/1422-0067/16/05/9341/s1.
