# Supplementary material for: Single Cell Confocal Raman Spectroscopy of Human Osteoarthritic Chondrocytes: A Preliminary Study
Source: Int J Mol Sci. 2015 Apr 24;16(5):9341–53. doi: 10.3390/ijms16059341 (PMC4463591; doi:10.3390/ijms16059341)
Supplement: Supplementary file 1 [file ijms-16-09341-s001.pdf]

Supplementary Information

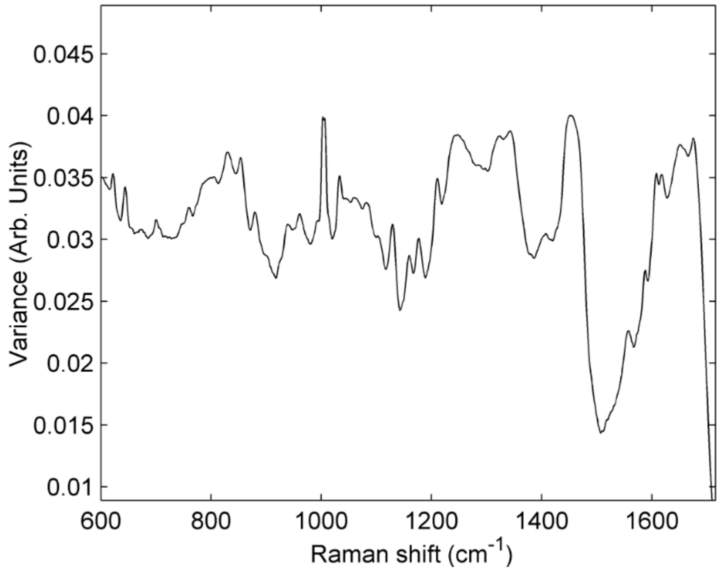

Figure S1. Plot of variance, between the 3 cell types, at each wavelength position of the Raman spectrum.

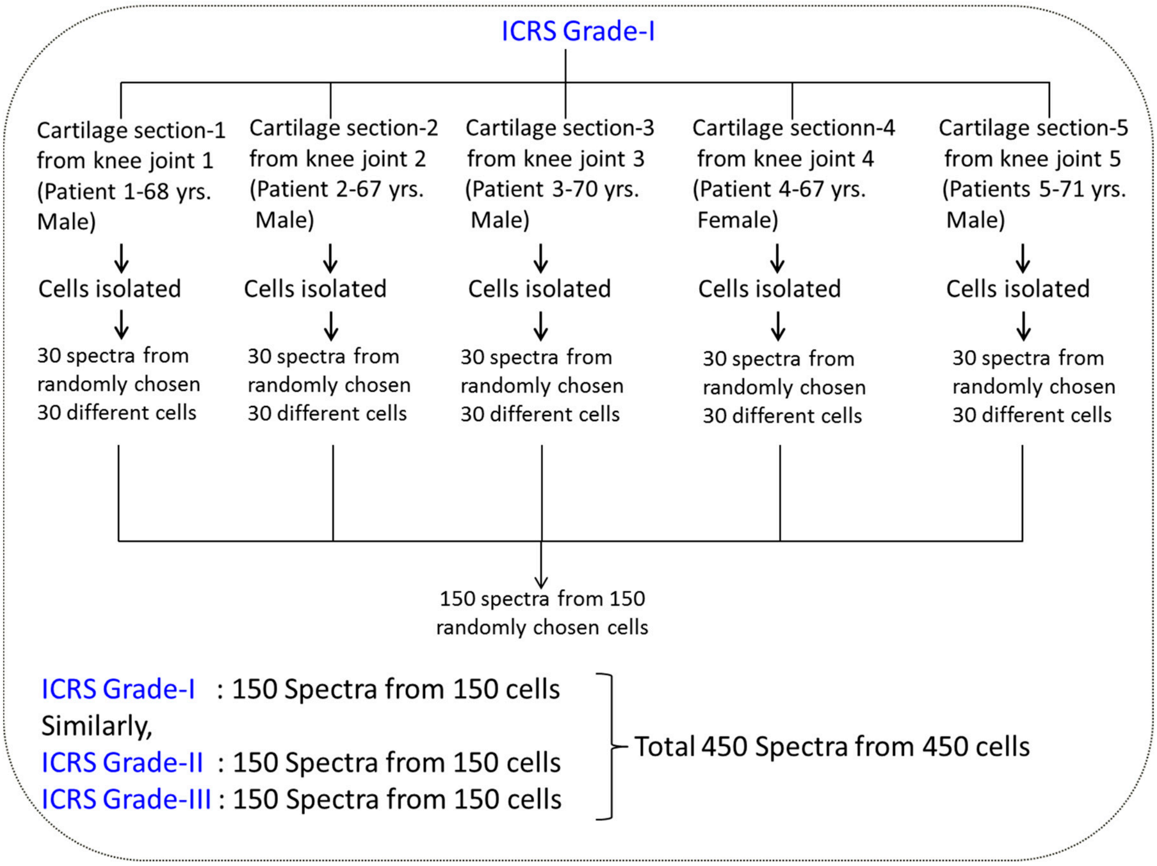

Figure S2. Classification of samples and acquisition of Raman spectra.

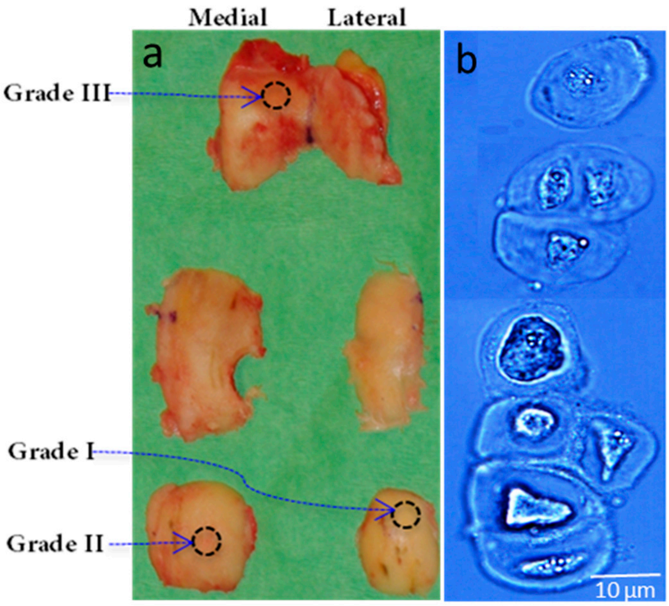

Figure S3. A typical image of (**a**) the representative area of osteoarthritic (ICRS Grade I, II and III) cartilage sections and (**b**) the representative bright-field micrographs of isolated human chondrocytes (cells) obtained from osteoarthritic (ICRS Grade-I) cartilage.

Table S1. ICRS grading based on the Outbridge score [38,39].

Grade	Property
I	Soft indentation, superficial lesions, fissures and cracks
II	Fraying, lesions extending down to <50% of cartilage depth
III	Cartilage defects extending down >50% of cartilage depth, loss of cartilage thickness
IV	Complete loss of cartilage thickness, bone only
